# Comparing Robot-Assisted and Laparoscopic Inguinal Hernia Repair: A Systematic Review and Meta-Analysis

**DOI:** 10.7759/cureus.60959

**Published:** 2024-05-23

**Authors:** Talal Khewater, Abdullah M Al Madshush, Mohammed I Altidlawi, Hamad Faya, Maryam Alanazi, Meshaal Mohammad M Alqahtani, Ibrahim A Alghamdi, Muhammad A Almotawa, Mohammed T Mirdad, Bandar A Alqahtani, Yasmeen Sleem, Rasha Mirdad

**Affiliations:** 1 Bariatric and Advanced Laparoscopic Surgery, King Salman Armed Forces Hospital, Tabuk, SAU; 2 College of Medicine, University of Tabuk, Tabuk, SAU; 3 Surgery, University of Tabuk, Tabuk, SAU; 4 Medicine, Faculty of Medicine, King Khalid University, Abha, SAU; 5 General Surgery, Faculty of Medicine, University of Tabuk, Tabuk, SAU; 6 College of Medicine, King Khalid University, Aseer, SAU; 7 College of Medicine, King Khalid University, Abha, SAU; 8 Surgery, King Khalid University, Abha, SAU; 9 Surgery, Armed Forces Hospital, Abha, SAU

**Keywords:** laparoscopy, laparoscopic inguinal hernia, robot-assisted laparoscopy, inguinal hernia repair, hernia

## Abstract

Inguinal hernia repair is a common surgical intervention. Advancements in minimally invasive techniques, specifically laparoscopic (LR) and robot-assisted (RR) approaches, have reshaped the landscape of surgical options. This meta-analysis aimed to systematically assess and compare the effectiveness and safety of laparoscopic and robot-assisted inguinal hernia repair through a comprehensive review of the literature. A systematic search of databases was conducted to identify relevant studies published up to November 30, 2023. Fifteen studies, encompassing a total of 64,568 participants, met the inclusion criteria. Pooled estimates for key outcomes, including duration of operation, overall complications, and surgical site infection (SSI), were calculated using random-effects models. This meta-analysis revealed a statistically significant difference in the duration of surgery, favoring laparoscopic repair over robot-assisted techniques (mean difference: 26.85 minutes, 95% CI (1.16, 52.54)). Overall complications did not significantly differ between the two approaches (odds ratio: 1.54, 95% CI (0.83, 2.85)). However, a significantly greater risk of SSI was identified for robot-assisted procedures (odds ratio: 3.32, 95% CI (2.63, 4.19)). This meta-analysis provides insights into the comparative effectiveness of laparoscopic and robot-assisted inguinal hernia repair. While laparoscopy has shorter operative times and comparable overall complication rates, the increased risk of SSI during robot-assisted procedures necessitates careful consideration in clinical decision-making. Surgeons and healthcare providers should weigh these findings according to patient characteristics, emphasizing a personalized approach to surgical decision-making. The evolving landscape of inguinal hernia repair warrants ongoing research to refine techniques and optimize outcomes for the benefit of patients undergoing these procedures.

## Introduction and background

Inguinal hernia repair is one of the most commonly performed surgical procedures worldwide, reflecting its prevalence and impact on the patient population [1,2]. An inguinal hernia occurs when abdominal tissues protrude through a weak spot or tear in the abdominal wall. This condition not only is a source of discomfort for affected individuals but also poses a considerable burden on healthcare systems worldwide [3,4].

Traditionally, open surgical techniques, such as the Shouldice or Bassini repairs, are the primary approaches for inguinal hernia repair [5,6]. While effective, these procedures often necessitate larger incisions, resulting in extended recovery times, increased postoperative pain, and a greater risk of complications [5]. The advent of minimally invasive techniques marked a paradigm shift in the field, offering patients and surgeons alternative approaches that aimed to mitigate these challenges [7].

Two notable minimally invasive techniques that have gained prominence in inguinal hernia repair are the laparoscopic and robot-assisted approaches [2]. Laparoscopic inguinal hernia repair, introduced in the 1990s, involves the insertion of a laparoscope and specialized instruments through small incisions, allowing real-time visualization and repair of a hernia with minimal disruption to surrounding tissues [8]. This technique has rapidly gained popularity due to its associated benefits, including reduced postoperative pain, shorter hospital stays, and faster recovery times [9,10].

The evolution of laparoscopic techniques has laid the foundation for further innovations in the form of robot-assisted surgery [11]. The introduction of robotic platforms, such as the da Vinci Surgical System (Intuitive Surgical, Inc., Sunnyvale, California, US), has led to enhanced dexterity, three-dimensional visualization, and improved ergonomics for inguinal hernia repair [11,12]. Surgeons operating these robotic systems can manipulate instruments with increased precision, facilitating intricate maneuvers in confined spaces [13].

Both laparoscopic and robot-assisted techniques aim to achieve the primary goals of inguinal hernia repair - effectively reducing the hernia, reinforcing the weakened abdominal wall, and minimizing postoperative morbidity [14]. The recurrence rate for laparoscopic inguinal hernia repair is 1.5%, compared to 2.7% for robot-assisted repair [14]. In terms of chronic postoperative inguinal pain, no significant difference in chronic postoperative pain between laparoscopic and robot-assisted inguinal hernia repair. When it comes to mesh infection, there is no significant difference in mesh infection rates between laparoscopic and robot-assisted inguinal hernia repair [6]. However, as these techniques have become integral parts of surgical practice, clinicians and researchers are facing the ongoing challenge of determining their comparative effectiveness and safety [2].

The selection of the most suitable technique for inguinal hernia repair involves a multifaceted consideration of various factors [1]. The choice between laparoscopic and robot-assisted approaches necessitates an understanding of their respective advantages, drawbacks, and outcomes [15]. Key factors under scrutiny include operative times, overall complications, and the incidence of surgical site infections (SSI). While shorter operative times may be indicative of procedural efficiency, an elevated risk of complications or infections could tip the balance in favor of one technique over the other [16].

The duration of the operation plays a significant role in patient care, affecting factors such as anesthesia exposure, operating room utilization, and postoperative recovery. Shorter operating times are generally associated with reduced intraoperative complications, decreased anesthesia-related risks, and improved patient satisfaction. Comparing the duration of laparoscopic and robotic procedures can help healthcare providers evaluate the efficiency and feasibility of each technique [13].

Assessing overall complications is essential for evaluating the safety and effectiveness of surgical interventions. Complications such as postoperative pain, wound infections, hernia recurrence, and nerve injuries can significantly impact patient recovery and long-term outcomes. By comparing the overall complication rates between laparoscopic and robotic inguinal hernia repair, healthcare providers can determine which approach is associated with better patient outcomes and fewer adverse events [15]. Surgical site infections (SSI) are a common and potentially serious complication following hernia repair surgery. Infection at the surgical site can lead to prolonged hospital stays, delayed wound healing, increased healthcare costs, and in severe cases, systemic infection and sepsis. Evaluating the incidence of SSI after laparoscopic and robotic procedures can help identify the safest and most effective technique for reducing the risk of postoperative infections and improving patient recovery [16].

As the landscape of inguinal hernia repair continues to evolve, the findings from this meta-analysis will contribute to the ongoing discourse surrounding the optimal surgical approach, ultimately striving to enhance patient outcomes and refine best practices in the field of hernia surgery. This meta-analysis aimed to compare robot-assisted and laparoscopic inguinal hernia repair in terms of the duration of operation, overall complications, and surgical site infection (SSI).

## Review

Methodology

Study Design

This systematic review and meta-analysis is reported in accordance with Preferred Reporting Items for Systematic Reviews and Meta-Analyses (PRISMA) guidelines [17]. The protocol for this meta-analysis was not registered. However, the methodology adheres to established guidelines and standards for conducting meta-analyses in the field of healthcare research.

Search Strategy

A systematic and exhaustive literature search was conducted to identify relevant studies comparing robot-assisted and laparoscopic inguinal hernia repair. Databases, including PubMed, Embase, and the Cochrane Library, were searched from inception to the date of the search (up to January 2023). The search terms included a combination of Medical Subject Headings (MeSH) terms and keywords related to “inguinal hernia,” “robot-assisted,” and “laparoscopic surgery.” The search strategy was developed with the assistance of a medical librarian to ensure comprehensiveness.

Study Selection

Two independent reviewers screened the initially identified studies based on predetermined inclusion and exclusion criteria. The inclusion criteria included studies comparing robot-assisted and laparoscopic inguinal hernia repair, including randomized controlled trials (RCTs) and prospective and retrospective cohort studies. The exclusion criteria included studies that do not directly compare these two procedures, focus on other hernia repair methods, involve pediatric populations, or are non-English studies, case reports, conference abstracts, and studies with insufficient data for meta-analysis.

Data Extraction

Data extraction was independently conducted by two reviewers using a standardized form. The extracted information included study characteristics (author, publication year, study design), participant demographics (sample size, age, sex distribution), and outcome data (duration of operation, overall complications, surgical site infection).

Data Synthesis

Quantitative data synthesis was performed using Review Manager software, version 5.4 (Cochrane Collaboration, London, UK). Fixed-effects and random-effects models were applied to estimate pooled effect sizes for continuous and binary outcomes, respectively. The inverse variance method was used for continuous outcomes. To assess the robustness of the findings, sensitivity analyses were performed by excluding studies with extreme effect sizes. Publication bias was evaluated using funnel plots for each outcome.

Statistical Analysis

Continuous outcomes, such as the duration of operation, are expressed as the mean difference with the corresponding 95% confidence interval (CI). Binary outcomes, including overall complications and surgical site infection, are presented as odds ratios (ORs) with 95% CIs.

Results

**Figure 1:**
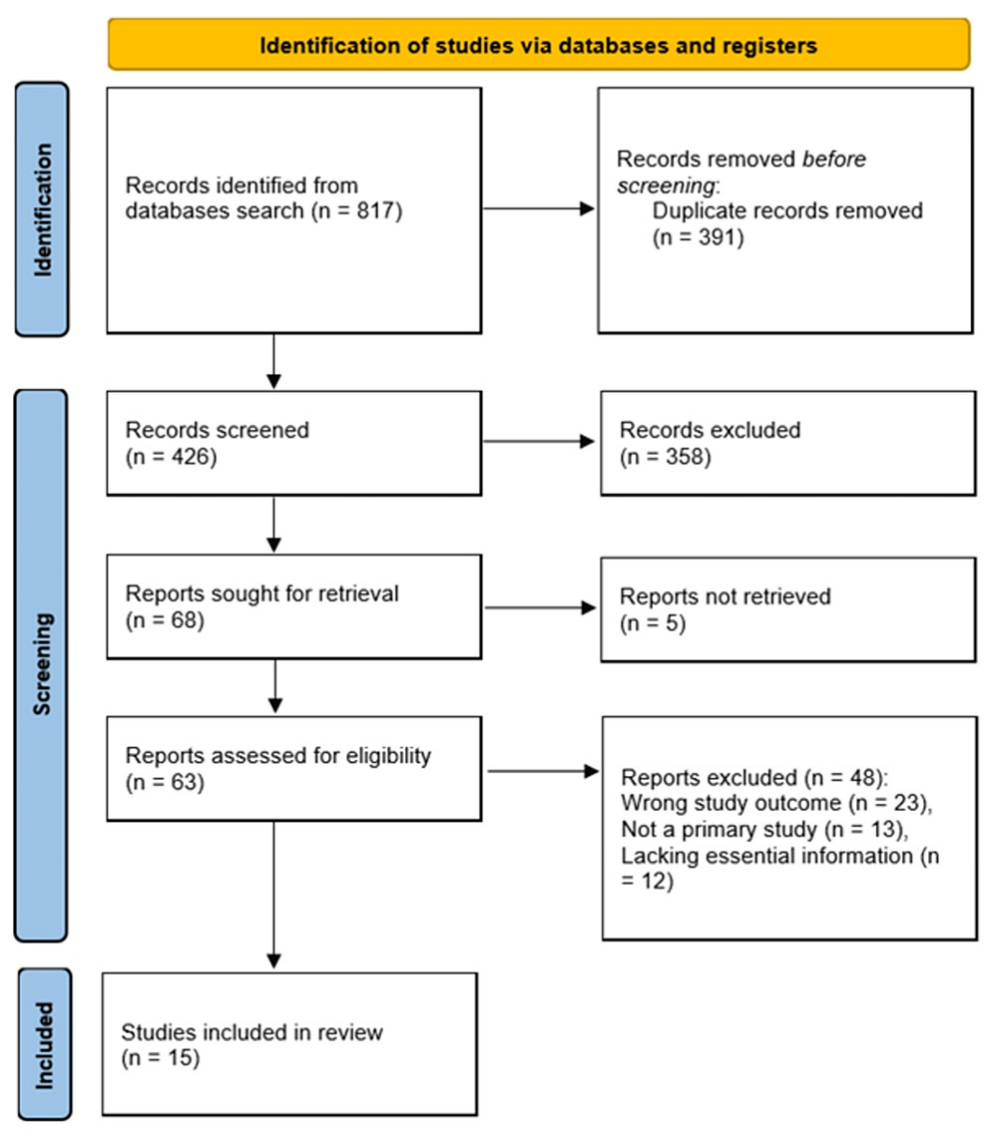
PRISMA flow diagram summarizing the study search and screening process PRISMA: Preferred Reporting Items for Systematic Reviews and Meta-Analyses

The initial database search yielded 817 records. After removing duplicates, 426 records were screened based on title and abstract. Subsequently, 358 studies were excluded for various reasons, such as irrelevant topics, inappropriate study designs, or insufficient data for meta-analysis. Of the remaining 63 studies, 5 did not have available full texts. Finally, 15 studies met the inclusion criteria and were included in the meta-analysis, as illustrated in Figure 1.

Study Characteristics

The characteristics of the 15 included studies are detailed in Table 1. The studies were published between 2014 and 2023 and originated from various countries, including the USA and Turkey. The total number of participants across all studies was 19,962, with individual study sample sizes ranging from 12 to 6,063.

**Table 1:** Characteristics of the included studies and populations (n = 15) NR: Not Reported

Study	Study design	Country	Study duration	Robot-assisted group				Laparoscopy group			
				Count	Age (years)	BMI (kg/m^2^)	Male (%)	Count	Age (years)	BMI (kg/m^2^)	Male (%)
Aghayeva et al. [18]	Retrospective single center	Turkey	2016–2018	43	52.1 ± 16.7	25.5 ± 3.5	93.1	43	52.3 ± 16.7	25.2 ± 2.8	93.1
Ayuso et al. [19]	Prospective single center	USA	2012–2020	141	58.6 ± 13.8	29.1 ± 21.0	NR	141	54.4 ± 15.5	27.1 ± 5.1	NR
Charles et al. [20]	Retrospective single center	USA	2012–2016	69	52 (39-62)	24.9 (22.9–28.7)	90	241	57 [45–67]	25.8 [23.1–28.4]	73
Higgins et al. [21]	Retrospective study	USA	2013–2015	12	NR	NR	NR	274	NR	NR	NR
Holleran et al. [22]	Retrospective multicenter	USA	2008–2019	6063	60.8 ± 12.2	29.5 ± 6.1	92.7	18035	60.3 ± 12.6	26.2 ± 4.2	99.5
Huerta et al. [23]	Retrospective study	USA	2005–2017	71	59.9 ± 12.5	27.5 ± 5.2	100	128	58.3 ± 12.4	26.3 ± 4.1	100
Khoraki et al. [24]	Retrospective single center	USA	2015–2017	45	49.6 ± 13.3	27.5 ± 5.8	93.3	138	50 ± 13.7	26.2 ± 3.6	96.4
Kudsi et al. [25]	Retrospective single center	USA	2012–2015	118	58.8 ± 15.4	28.44 ± 5.02	85.6	157	55.1 ± 14.8	27.01 ± 4.86	94.9
Kudsi et al. [26]	Retrospective single center	USA	2012–2020	547	59.3 ± 16	26.6 (24.2–29.8)	91.4	606	58.4 ± 15.8	26.4 (24–29.3)	93.7
LeBlanc et al. [27]	Prospective multicenter study	USA	2016–2018	80	58.95 (46.7–68.6)	27.1 (24.5–29.15)	95	80	59.7 (49.5–68.55)	26.8 (24.4–29.0)	92.5
Muysoms et al. [28]	Prospective single center	USA	2013–2017	49	60.4 (16.5)	25 (3.4)	97.9	64	59.0 (11.8)	24 (3.0)	96.9
Pokala et al. [29]	Retrospective multicenter	USA	2013–2017	594	NR	NR	95.3	540	NR	NR	80.4
Prabhu et al. [30]	Prospective randomized trial, multicenter	USA	2016–2019	48	56.1 (14.1)	24.9 (3.24)	88.9	54	57.2 (13.3)	26.9 (4.42)	91.67
Tatarian et al. [31]	Retrospective multicenter	USA	2010–2016	559	61.15 ± 11.55	NR	90.2	35565	53.44 ± 15.15	NR	92.9
Waite et al. [32]	Retrospective study	USA	2012–2014	39	58.1	27.5	97.4	24	57.5	27.6	100

The age and body mass index (BMI) of participants across the studies exhibited considerable variability. Ayuso et al. [19] reported a mean age of 58.6 years and a BMI of 29.1 kg/m², whereas Aghayeva et al. [18] observed a slightly younger cohort with a mean age of 52.1 years and a BMI of 25.5 kg/m². Charles et al. reported age as a range (52 (39-62)) and BMI as a range (24.9 (22.9-28.7)), reflecting the heterogeneity in participant characteristics [20].

The distribution of male participants varied across studies, with some studies, such as Aghayeva et al. [18], Ayuso et al. [19], Charles et al. [20], Huerta et al. [23], Khoraki et al. [24], Kudsi et al. [25], Kudsi et al. [26], LeBlanc et al. [27], Muysoms et al. [28], Prabhu et al. [30], Tatarian et al. [31], and Waite et al. [32], reporting percentages above 90%, indicating a predominance of male participants.

The included studies exhibited diverse designs, including retrospective single-center studies [18,20,23-26,32], prospective single-center studies [19,28,30], retrospective multicenter studies [21,29,31], and prospective multicenter studies [27]. Additionally, a prospective randomized trial with a multicenter design was conducted by Prabhu et al. [30].

Duration of Operation

**Figure 2:**
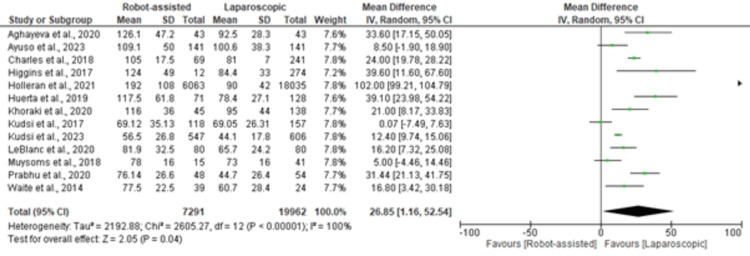
Forest plot of the surgical duration for robot-assisted versus laparoscopic inguinal hernia repair [18-28,30,32]

The Forest plot in Figure 2 presents the results for the surgical duration in robot-assisted versus laparoscopic inguinal hernia repair. The pooled estimate, based on 7291 robot-assisted cases and 19,962 laparoscopic cases, revealed a statistically significant difference in favor of laparoscopic repair, with a mean difference of 26.85 minutes (95% CI (1.16, 52.54)). Individual studies have consistently demonstrated shorter operative times for laparoscopic procedures.

Overall Complications

**Figure 3:**
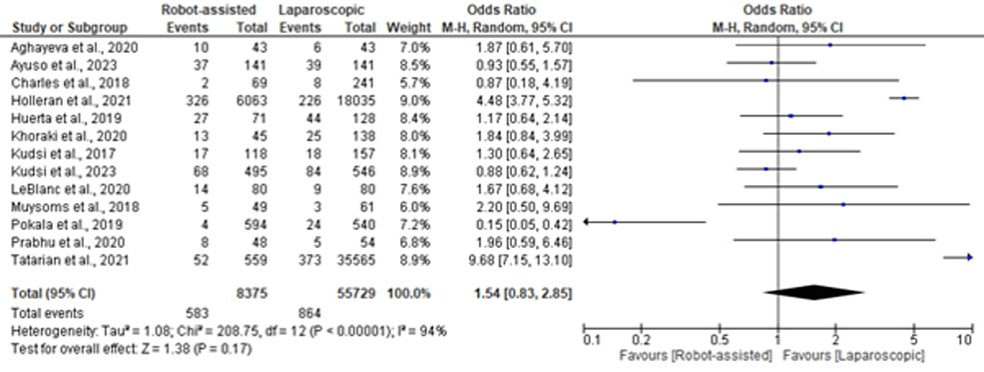
Forest plot of overall complications in robot-assisted versus laparoscopic inguinal hernia repair patients [18-31]

Figure 3 displays the forest plot for overall complications. The analysis, which included data from 8375 robot-assisted cases and 55,729 laparoscopic cases, did not reveal a statistically significant difference between the two approaches (OR 1.54, 95% CI (0.83, 2.85)). Individual study findings aligned with the overall pooled estimate, emphasizing the comparable safety profiles of robot-assisted and laparoscopic inguinal hernia repair.

Surgical Site Infection (SSI)

**Figure 4:**
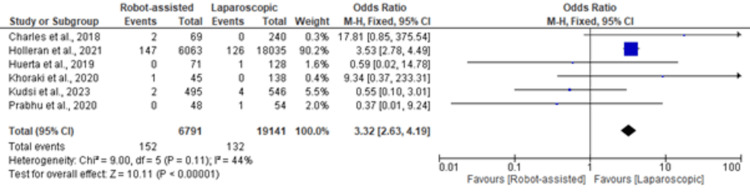
Forest plot of surgical site infection in robot-assisted versus laparoscopic inguinal hernia repair [20,22-25,30]

The Forest plot in Figure 4 depicts the results for surgical site infection (SSI). The analysis, which included 6,791 robot-assisted surgeries and 19,141 laparoscopic surgeries, revealed a significantly greater risk of SSI associated with robot-assisted repair (OR 3.32, 95% CI (2.63, 4.19)). While individual studies have reported varying SSI rates, the overall meta-analysis highlighted a consistent trend toward increased SSI risk with robot-assisted procedures.

The test for overall effect in the meta-analysis of duration of operation yielded a Z value of 2.05 (P = 0.04), indicating a statistically significant difference. The overall effect tests for overall complications (Z = 1.38, P = 0.17) and SSI (Z = 10.11, P < 0.00001) did not reach statistical significance.

Heterogeneity and Sensitivity Analysis

Heterogeneity was observed in the analyses for the duration of operation, overall complications, and SSI. Sensitivity analyses were performed to assess the robustness of the findings by excluding studies with extreme effect sizes. A sensitivity analysis was conducted by excluding the study conducted by Pokala et al. [29]. The exclusion of this study resulted in a notable change in the pooled estimates for overall complications. The updated pooled estimate became statistically significant (total events: 579; total participants: 7781; odds ratio: 1.86; 95% CI (1.03, 3.36); Z = 2.04; P = 0.04). This change highlights the influence of Pokala et al. [29] on the initial nonsignificant findings. The sensitivity analysis underscores the potential impact of individual studies on the overall results and emphasizes the need for cautious interpretation.

Publication Bias

**Figure 5:**
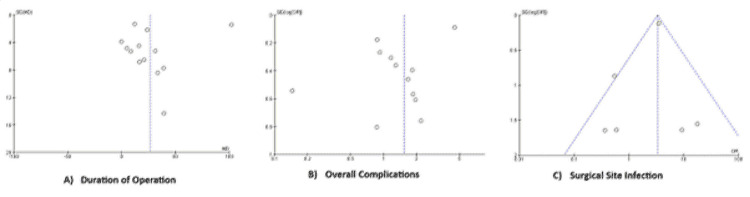
Funnel plots of the A) surgical duration, B) overall complications, and C) surgical site infections for assessment of publication bias.

Funnel plots for the duration of the operation, overall complications, and incidence of SSI are presented in Figure 5. Visual inspection of the plots indicated a more-or-less central distribution. Egger's test and Begg's test did not reveal significant asymmetry in the funnel plots. The trim-and-fill method was not applied because no substantial publication bias was identified.

Discussion

Inguinal hernia repair, a common surgical procedure, has undergone transformative advancements with the introduction of robot-assisted and laparoscopic techniques [33]. As these approaches continue to evolve, understanding their comparative effectiveness and safety is crucial for informing clinical decision-making [34]. Our meta-analysis systematically examined 15 studies, encompassing 64,568 cases, to compare robot-assisted and laparoscopic inguinal hernia repair in terms of surgical duration, overall complications, and surgical site infection (SSI).

Our meta-analysis revealed a statistically significant difference in the duration of surgery, favoring laparoscopic repair. The pooled estimate indicated that laparoscopic procedures were, on average, 26.85 minutes shorter than robot-assisted techniques (95% CI (1.16, 52.54)). These findings align with those of the individual studies included in the analysis, emphasizing the efficient and expeditious nature of laparoscopic inguinal hernia repair.

This result underscores the importance of considering the operation time as a critical factor in surgical decision-making [35]. The reduced duration not only contributes to potential cost savings but also aligns with the broader trend in minimally invasive surgery, emphasizing quicker recovery times and reduced postoperative morbidity [33,35].

In contrast to the observed difference in operative time, our meta-analysis did not reveal a statistically significant difference in overall complications between robot-assisted and laparoscopic approaches. The aggregated data, incorporating 64,104 patients, underscore the comparable safety profiles of both techniques. These findings are consistent with those of individual studies, including those of Charles et al. [20] and Prabhu et al. [30], and contribute to the overall equivalence of complication rates.

The absence of a significant difference in overall complications implies that both robot-assisted and laparoscopic approaches can be considered safe and effective for inguinal hernia repair. When conducting the sensitivity analysis, we excluded the study of Pokala et al. [29], and the pooled estimate became statistically significant.

Notably, our meta-analysis revealed a significantly greater risk of SSI associated with robot-assisted inguinal hernia repair. The odds ratio of 3.32 (95% CI (2.63, 4.19)) suggested a threefold increased risk of SSI in robot-assisted procedures compared to laparoscopic techniques. This finding is noteworthy and warrants careful consideration in clinical decision-making [36,37].

The increased risk of SSI in robot-assisted procedures may be attributed to various factors, including longer operative times, increased complexity of the robotic approach, and variations in the learning curves associated with these techniques [38,39]. However, further research is needed to determine the specific factors contributing to the observed difference in SSI rates.

The findings of this meta-analysis hold significant implications for both clinicians and patients facing the decision between robot-assisted and laparoscopic inguinal hernia repair. The shorter operative times associated with laparoscopy align with the broader goals of minimally invasive surgery, emphasizing patient-centric outcomes such as faster recovery and reduced postoperative discomfort [2,33,36].

However, the increased risk of SSI in robot-assisted procedures raises concerns and necessitates a nuanced approach. Surgeons should carefully weigh the potential benefits of shorter operative times against the increased infectious risks associated with robotic techniques [38]. Strategies to mitigate SSI risks in robot-assisted procedures, such as refined surgical techniques, improved training protocols, and strict adherence to infection control measures, should be explored and implemented [40].

Future research should focus on prospective studies with standardized protocols to minimize bias and enhance the comparability of the results. However, further investigations into the specific factors contributing to SSI during robot-assisted procedures are warranted. Additionally, long-term outcomes and cost-effectiveness analyses should be explored to provide a comprehensive understanding of the broader clinical and economic implications of these surgical approaches.

## Conclusions

In conclusion, our meta-analysis provides insights into the comparative effectiveness and safety of robot-assisted and laparoscopic inguinal hernia repair. While laparoscopy offers a time-efficient alternative with comparable overall complication rates, the increased risk of SSI in robot-assisted procedures necessitates careful consideration. Surgeons and healthcare providers should weigh these findings according to patient characteristics, emphasizing a personalized approach to surgical decision-making. Laparoscopic surgery may be more beneficial for patients with certain comorbidities, such as obesity or diabetes, due to its minimally invasive nature and shorter recovery times. On the other hand, robotic surgery may be more appropriate for patients with complex anatomical structures or tumors that are difficult to access with traditional laparoscopic techniques. Additionally, factors such as patient preferences, surgical experience, and hospital resources should also be taken into consideration when deciding between laparoscopic or robotic surgery. By taking a personalized approach to surgical decision-making, healthcare providers can optimize patient outcomes and improve overall satisfaction with their care. The evolving landscape of inguinal hernia repair warrants ongoing research to refine techniques and optimize outcomes for the benefit of patients undergoing these procedures.
